# The Impact of Surface Charges of Carboxylated Cellulose Nanofibrils on the Water Motions in Hydrated Films

**DOI:** 10.1021/acs.biomac.1c01517

**Published:** 2022-07-05

**Authors:** Valentina Guccini, Shun Yu, Zhoujun Meng, Eero Kontturi, Franz Demmel, Germán Salazar-Alvarez

**Affiliations:** † Department of Materials and Environmental Chemistry (MMK), Stockholm University, Stockholm SE-10691, Sweden; ‡ Department of Bioproducts and Biosystems, School of Chemical Engineering, Aalto University, P.O. Box 16300, Aalto 00076, Finland; § Smart Materials, Division of Bioeconomy and Health, RISE Research Institute of Sweden, Drottning Kristinas väg 61, Stockholm 114 86, Sweden; ∥ ISIS Facility, Rutherford Appleton Laboratory, Didcot OX11 0QZ, UK; ⊥ Department of Materials Science and Engineering, Ångström Laboratory, Uppsala University, Box 35, Uppsala SE-751 03, Sweden; # Center for Neutron Scattering, Uppsala University, Box 35, Uppsala SE-751 03, Sweden

## Abstract

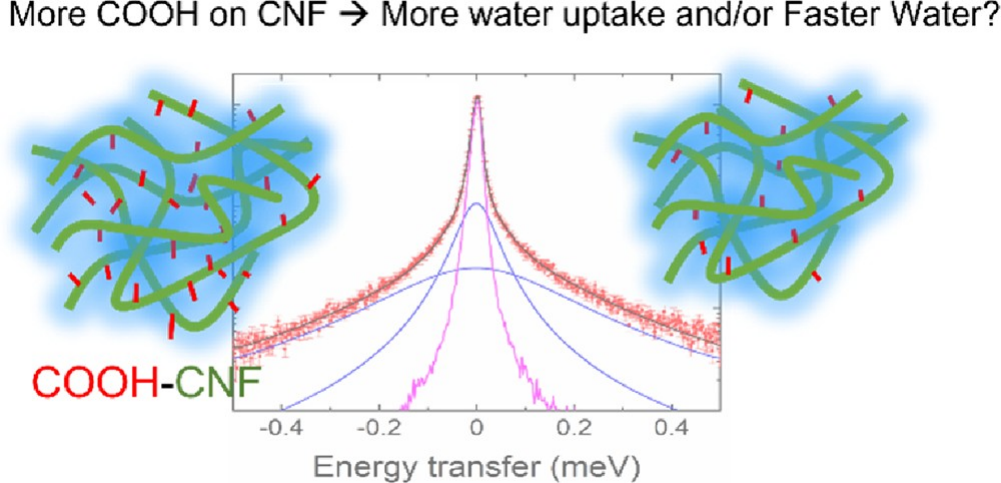

Cellulose nanofibrils (CNFs) with carboxylated surface ligands are a class of materials with tunable surface functionality, good mechanical properties, and bio-/environmental friendliness. They have been used in many applications as scaffold, reinforcing, or functional materials, where the interaction between adsorbed moisture and the CNF could lead to different properties and structures and become critical to the performance of the materials. In this work, we exploited multiple experimental methods to study the water movement in hydrated films made of carboxylated CNFs prepared by TEMPO oxidation with two different surface charges of 600 and 1550 μmol·g^–1^. A combination of quartz crystal microbalance with dissipation (QCM-D) and small-angle X-ray scattering (SAXS) shows that both the surface charge of a single fibril and the films’ network structure contribute to the moisture uptake. The films with 1550 μmol·g^–1^ surface charges take up twice the amount of moisture per unit mass, leading to the formation of nanostructures with an average radius of gyration of 2.1 nm. Via the nondestructive quasi-elastic neutron scattering (QENS), a faster motion is explained as a localized movement of water molecules inside confined spheres, and a slow diffusive motion is found with the diffusion coefficient close to bulk water at room temperature via a random jump diffusion model and regardless of the surface charge in films made from CNFs.

## Introduction

Cellulose nanofibrils (CNFs) packed via cellulose polymer chains are building blocks for cellulose fibers, which are further integrated into the plant cell walls,^1^ playing a central role in maintaining the mechanical strength of the plant body. CNFs could be extracted from the raw cellulose fibers as colloidally stable nanofibrils by using a combination of chemical and mechanical treatments. The obtained materials do not only preserve the mechanical strength but also demonstrate a chemical versatility enabled by a wide portfolio of chemical modifications on the hydroxyl groups present at their surface.^2,3^ CNFs have become a popular material platform for developing novel composites,^2−7^ and CNF-based materials have demonstrated a wide range of morphologies such as hydrogel/foam,^5,8^ fibers,^9,10^ membranes,^11−13^ and additives,^14−16^ often characterized by a hierarchical structure. A commonly used extraction method is conducted via 2,2,6,6-tetramethylpiperidine-1-oxyl (TEMPO)-mediated oxidation and mechanical fluidization.^17,18^ The prepared carboxylated CNFs have a high aspect ratio, e.g., cross section of ca. 5 nm and over micrometers long,^5^ and activated carboxylate surface ligands.^17^ The materials made of carboxylated CNFs have demonstrated excellent mechanical properties,^9,10,19^ and this type of CNF could also be used as carriers to load other functional materials in optic,^20^ electronic,^21^ and energy devices.^22^

Despite many promising applications, use of CNFs must cope with the complex cellulose–water interaction, which is one of the most important scientific topics in the field of wood-based materials science. Water adsorption to cellulose crystals is dominated by enthalpic contributions.^23,24^ At the cellulose surface, the primary site of interaction is the hydroxyl groups of cellulose, which may interact with water via hydrogen bonding according to their accessibility.^25^ In particular, the hydroxyls in positions 2 and 6 of the cellulose ring structure are more susceptible to moisture absorption than the one in position 3, which has a predominant role in stabilizing the cellulose structure.^26^ At the nanoscale, CNFs are recognized as being highly crystalline with small segments of non-crystalline regions in the longitudinal direction. These areas were formerly called ″amorphous regions″ but modern accounts strongly suggest that they are too short to be consisting of bulky, amorphous cellulose as their length is allegedly somewhere between 1 and 5 nm.^27,28^ Unlike the completely disordered cellulose polymer chains, which are more accessible to water molecules,^29^ the disordered segments do not likely contribute much to the water uptake of CNFs but they are more susceptible to certain chemical reactions, notably acid hydrolysis.^30^ The crystalline phase of CNFs is on the other hand the most compacted crystalline building blocks with practically no access to water infiltration and is also resistant to hydrolysis.^31^ Nevertheless, the CNFs can further assemble or entangle into CNF aggregates, resulting in confined spaces that affect the characteristics of water trapped insides. With an increased water uptake, the crystalline phase may deform,^32^ resulting in a lattice constant change. The water–cellulose interaction accumulates till the macroscale, where not only does the capillary force of the water play an important role as it influences the gas and liquid exchange but also the mechanical properties, such as wet strength, become greatly influenced.^33^ In this respect, CNF–water interactions are crucial as they can represent either a detriment or an advantage depending on applications. For instance, the hydroxyl groups dominate the CNF chemistry and together with the high surface-to-volume ratio enable high water uptake, which can hinder the strength and stiffness of load-bearing CNF-based materials.^34−36^ On the other hand, the combined excellent mechanical properties of CNFs and strong water affinity are a unique asset in applications such as biocompatible matrixes and cell growth^37−41^ or polymeric electrolyte membranes (PEMs) for fuel cells.^42^ In the case of the PEMs produced from carboxylate CNFs and sulfate cellulose nanocrystals (CNCs), they could outperform the bench market material (Nafion) in terms of stability of the proton conductivity at various relative humidities (between 65 and 95 RH% and 30 °C)^11^ and high temperature.^43^ Thus, knowing the water dynamics within CNF matrixes will certainly contribute to the design of materials for these relevant applications.

The dynamics of water in cellulose matrixes have been studied by several techniques at different spatial and time scales. Dielectric spectroscopy has been used to probe the orientational motions of the hydroxyl groups in a time frame from 1 μs to 10 s.^44−46^ IR spectroscopy has been used to study the dynamics of water adsorption on cellulose fiber surfaces via hydrogen bonding.^26,47^ The water diffusion coefficient is an important parameter often used to describe how water moves in the cellulose. In many cases, two-component diffusion models are proposed. However, “two components” were mainly inferred from the experimental observations and may differ depending on the material system, measurement techniques, and moisture conditions.^25,48,49^ Using NMR spectroscopy, Topgaard and Söderman studied the cellulose fibers in filter paper with low water contents in the range of 10–20% (*m*_water_/*m*_cellulose_) at 25 °C.^49^ They report that the water diffusion coefficient parallel to the paper plane is found mainly to be continuously distributed from 10^–11^ to 10^–10^ m^2^/s (i.e., 10^–7^ to 10^–6^ cm^2^/s) regardless of the detailed interaction, and at a water content of 10%, nearly 46% of the water took part in hydration, resulting in a slow motion. Lindh et al.^25^ observed, using ^2^H NMR, that in cellulose microfibril aggregates, e.g., a few closely packed CNFs, there are two types of water: mobile water molecules on the surface of microfibril aggregates and trapped immobilized water within the microfibril aggregates. In this case, the surface water becomes more movable upon increasing hydration while the confined water is only marginally influenced by the hydration where limited water–water contacts hinder the molecular motions for a further increase.^25^ Perkins and Batchelor used a NMR pulsed field gradient to investigate different celluloses including CNFs produced by mechanically refined Northern (Canadian) Softwood Bleached Kraft, and they found that two-component diffusion models, i.e., fast and slow components are valid for all materials, with the slow components as an inner layer adjacent to the cellulose surface and shielding the faster water, which are further away from the cellulose surface.^48^ In their work, the fastest water diffusion coefficient in CNF is around 1.0 × 10^–9^ m^2^/s (i.e., 1.0 × 10^–5^ cm^2^/s) at 20 °C.^48^ In the wood matrix, where the cellulose and water interact in a complex hierarchical morphology, free water does not show any significant difference in mobility and thermal behavior compared to regular water, which is mainly because of the water in the wood cell lumen.^50^ To study the water dynamics, neutron scattering provides a complementary perceptive by evaluating the energy and momentum transfer of water, thus giving information about the spatial (Å to nm) and temporal scale (ps to ns). Most published works are carried out on large cellulose fibers.^51−54^ Recently, O’Neill et al.^51^ have used QENS at BASIS, the Backscattering Spectrometer, at the ORNL Spallation Neutron Source (SNS) in an energy range of +/–100 μeV to study the dynamics of water bound to partially deuterated highly crystalline bacterial celluloses, which are characterized as a “mesoporous material” consisting of cellulose microfibrils with 5 nm width and ∼2 nm interfibrillar spaces. They also reported two types of water motion and that the water diffusion coefficient in their systems was 0.85 ± 0.04 × 10^–10^ m^2^ s^–1^ at 250 K and increased to 1.77 ± 0.09 × 10^–10^ m^2^ s^–1^ at 265 K, with residence time longer than 100 ps via a jump diffusion model.

As most nanostructures show different properties compared to their bulk counterparts, CNFs and their derived bulk structure have demonstrated very different properties compared to native bacterial cellulose and wood pulp.^3,38,42,55^ In spite of much work of NMR investigation on water dynamics in cellulose and CNF at room temperature and its vast technological importance,^25,48,49,56^ the temperature-dependent water dynamics of the CNF system is still scarcely investigated at the nanoscale to our knowledge.

In this work, we choose to study the water dynamics at different temperatures within hydrated films made of randomly oriented carboxylated CNFs, prepared by TEMPO-mediated oxidation^17^ via quasi-elastic neutron scattering (QENS) in combination with adsorption investigation via quartz crystal microbalance with dissipation (QCM-D) and morphological characterization via small-angle X-ray scattering (SAXS). Using QCM-D, we intend to estimate the contribution of water vapor on a single fibril level depending on relative humidity and the CNFs’ surface charge (carboxylate content). The CNFs with Na^+^ as a counterion (Na^+^-CNF) have better dispersibility than protonated CNFs (H^+^-CNF), allowing their preparation as separate single fibrils over the sensor surface of QCM-D. By comparing the difference of the water-uptaken CNF in different formats, the film morphology clearly plays an important role in the storage of water between fibrils, the detailed geometry and impact of which to water dynamics are further investigated via SAXS and QENS. For SAXS and QENS, we focused on H^+^-CNF, which has been used for polymeric electrolyte membranes^11^ and the water dynamics of which are critical. SAXS was used to observe the morphological changes of the films upon moisture absorption, to which both the CNF network characteristics and surface charge contributes. The water dynamics depending on the temperature and moisture contents were studied using QENS in the “IRIS” Time-of-flight (TOF) inverted-geometry crystal analyzer spectrometer at the ISIS neutron facility, UK, and correlated with the nanofibril carboxylate content. Our study offers a model case of the water dynamics that characterized CNF-based materials at a faster time domain, which is relevant for a wide range of applications. Given the fact that our CNF films are analogous or similar to those successfully used as polymeric electrolyte membranes,^11,42,43^ the findings on the relation between CNF surface charge roles and the proton conduction mechanism in CNF-based polymeric electrolyte membranes will directly contribute to developing more efficient and environmentally compatible energy devices. Furthermore, understanding the dynamics of CNF–water interactions can help to find innovative ways to mitigate the detrimental effect of water on the wet strength of load-bearing CNF-based materials or applications that require anhydrous conditions (e.g., lithium ion batteries).

## Materials and Experimental Methods

### Preparation of the CNF Films

**Scheme 1:**
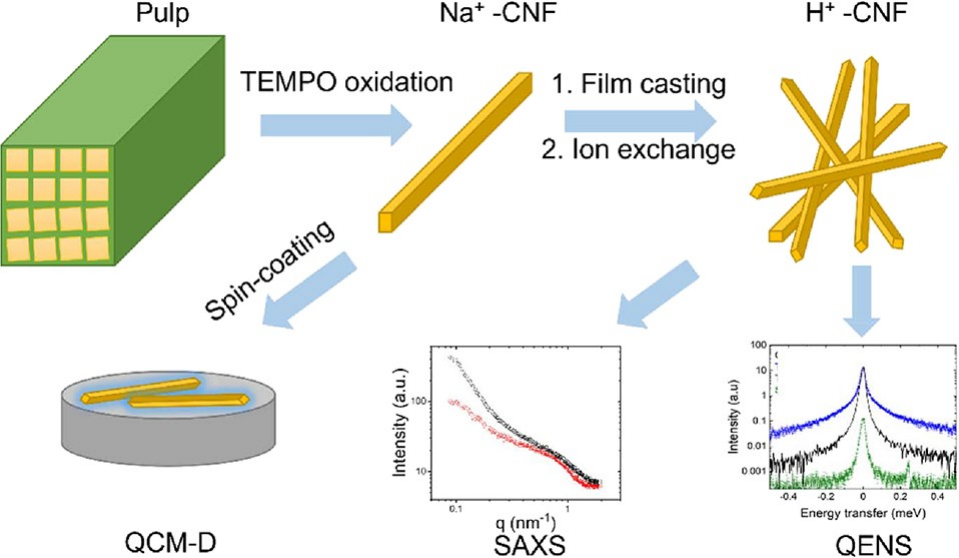
Material Preparation Procedure and Characterization

Carboxylated CNFs with surface charges of 600 and 1550 μmol g^–1^ were prepared from the never-dried cellulose pulp supplied by Domsjö Fabriker AB (Domsjö, Sweden). The materials are prepared by following the same protocols of our previous work in ref (11) and the preparation procedure and characterization are summarized in Scheme 1. The pulp was washed with a solution of HCl at pH 2 and then processed via TEMPO-mediated oxidation based on the procedure of Saito et al.^57^ Na^+^-CNF films were prepared via drop casting of the CNF suspensions (0.3 wt %) in polystyrene Petri dishes with a diameter of 5.5 cm. The suspensions were dried at 50% RH and 30 °C for 48 h. After the samples were dried, the counterions of the carboxylate groups were exchanged from sodium to hydrogen by immersing the films in a solution of 0.01 M sulfuric acid for 30 min and rinsing them in Milli-Q water until the pH of the washing water was neutral, producing protonated CNF (H^+^-CNF).^58^ All specimens made of H^+^-CNF were conditioned in a humidity chamber; the ones with high moisture content were conditioned at 95% RH and 30 °C over 24 h and named CNF600-95% and CNF1550-95%, whereas the sample with lower moisture content (CNF1550-50%) was conditioned at 50% RH at 30 °C over 24 h. The dry reference sample was conditioned at 10% RH and 30 °C over 48 h and named CNF600-Dry. Their moisture content *w*%(RH) was estimated via a gravimetric method via a balance with a 10 mg precision, comparing the mass of each sample equilibrated at 10% RH (*W*_dry_) with that at the specific RH (*W*_RH_), i.e., *w* % (RH) = (*W*_RH_ – *W*_dry)_/*W*_dry_ × 100.

### QCM-D Characterization

Before spin-coating, the QCM-D sensors were cleansed in a UV ozone cleaner (Bioforce Nanosciences Inc., CA, USA) for 20 min. The Na^+^-CNF suspensions were diluted to 0.01 wt % and spin-coated on gold QCM-D sensors using a WS-650SX-6NPP/LITE (Laurell Technologies Corporation, North Wales, PA, USA), at 4000 rpm for approximately 90 s. The measurements were carried out using an E4 instrument (Q-Sense, AB, Gothenburg, Sweden) equipped with a QHM 401 humidity module. The areal mass (Δ*m*) of the spin-coated CNFs was determined as described by Peresin et al.^59^ by measuring the frequency response (Δ*f*) in air (normal atmospheric conditions) before and after film deposition on the QCM-D sensors. To start, the CNF samples were allowed to equilibrate at 11% relative humidity (RH) for approximately 18 h by passing a saturated solution of LiCl through the module at a rate of 50 μL·min^–1^. After this equilibration step, water vapor adsorption experiments were carried out in five steps by gradually increasing the RH within the chamber using five different saturated salt solutions (Figure S1) at a rate of 50 μL·min^–1^ for 20 min at 23 °C, reaching RH values of 11, 33, 53, 75, and 97%. Once this cycle was complete, the system was washed with Milli-Q water, which will give each cycle the same starting state and avoid hysteresis if any. Prior to the mass change determination, each sample was dried with nitrogen at 80 °C in an oven for 15 min. The collected frequency data were then stitched together using QTools software, and the areal mass was calculated according to the Sauerbrey equation:^60^
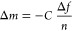
1where Δ*f* = *f* – *f_0_* is the resonance frequency, *n* is the overtone number (*n* = 3, 5, 7, ...), and *C* is the sensitivity constant of the sensor (*C* ≈ 0.177 mg·m^–2^·Hz^–1^).

### SAXS Characterization

The SAXS measurements were performed at beamline P03 “MiNaXS” of PETRA III storage ring at the Deutsches Elektronen-Synchrotron (DESY), Germany, by following the same protocols as our previous work.^11^ The wavelength of the incident X-ray is 0.0957 nm, and a beam size on the sample of about 20 × 20 mm^2^ was used. The sample-to-detector distance was calibrated by dry rat-tail collagen to 2500 ± 0.1 mm. The scattering patterns were recorded via a 2D pixel detector (Pilatus 1M from Dectris AG). The CNF films were cut into strips and stored at specific RH for SAXS measurement. The exposure time is determined to be 0.5 s by considering the X-ray radiation damage, which was carefully checked by comparing the scattering intensity change from a series of fast acquisitions. The scattering profile is an average of seven measurements at different positions. The *I*(*q*) at 95% RH were normalized by the Porod invariant *Q* to compensate for the change in scattering intensity, *I*, due to the variation of the CNF volume fraction, φ, and to highlight the structural information. *Q* = ∫_0_^∞^*q*^2^*I*(*q*)d*q* = 2π^2^Δρ^2^φ(1 – φ), where Δρ is the electron density difference between CNF and water.

### QENS Characterization

For the QENS measurements, the conditioned films were stacked together and sealed in a plate-like aluminum sample holder via an In–Sn alloy wire as the gasket until the QENS measurements. The measurements were performed with the “IRIS” time-of-flight (TOF) inverted-geometry crystal analyzer spectrometer at the ISIS neutron facility, UK. A total of 51 detectors were binned down to 25 groups, covering the range of transferred wave vectors (*Q*) from 0.4 to 1.8 Å^–1^. We used the PG002 reflection of the analyzer, which resulted in an energy resolution of Δ*E* = 0.0175 meV (FWHM). For a single spectrum, data for about 4 h of beamtime were collected. About 0.3 g of CNF films was placed into a flat aluminum sample cell. The cell was installed in a cryofurnace in a 45° orientation relative to the incoming beam. This orientation of the cell leaves a range of wave vectors around *Q* ∼ 1.7 A^–1^ difficult to analyze because in this scattering angle, the scattered neutrons have to pass a large part of the sample to reach the detector. The monitored normalized spectra were converted to energy and then re-binned in energy steps of 0.002 meV within an energy range of ±0.5 meV. A vanadium standard was measured for the efficiency calibration of the detectors. Measurement at a low temperature of CNF600-95% of 20 K was used as a resolution function for the data analysis (Figure S2A). The contribution from the empty can is negligibly small. The data reduction and analysis fitting were carried out via the software framework Mantid.^61^ In general, the motion of water molecules might consist of two parts, a translational diffusive motion and a localized motion, which might stem from rotational movements. The localized motion will be accompanied by an elastic contribution to the spectra. From the wave vector dependence of the width, the character of the motion can be inferred. When the width goes to zero for small wave vectors, the motion belongs to a translational diffusive motion and a non-zero value for the width for *Q* → 0 indicates a localized motion. The amplitude of the elastic intensity is called the elastic incoherent structure factor, EISF, and describes the geometry of the localized motion. Therefore, we have chosen the following approximate fit model for the spectra:
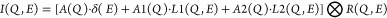
2where *A*(*Q*) is the elastic incoherent structure factor (EISF); δ(*E*) depicts the elastic scattering without energy transfer. *A*1(*Q*) and *A*2(*Q*) are the amplitudes of the quasi-elastic broadening, which consist of the following Lorentzian function:
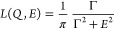
3in which Γ is the half-width at half maximum (HWHM). This model function is convoluted with the measured resolution function *R*(*Q*,*E*), which has been obtained through a low-temperature measurement. No background was added in the fit model eq 2. This is a minimal model to describe the motions of water molecules in confinement. The addition of a wave vector and temperature-dependent background would increase the number of fit parameters and might interfere with the fit procedure. Part of the vibrational density of states of the CNF film is already included in the fit because we are utilizing as resolution the low-temperature measured data. Figure S2A shows that the resolution function is identical to the dry film 250 K data and hence potential contributions from vibrational densities of states are included in the fit model. For fitting the data, the model in eq 2 was convoluted with the measured resolution function spectrum dependent. For the fit procedure, a Bayesian statistical approach was used, which provides more stable fit results compared to a Levenberg–Marquardt-based approach. Further short runs over a wide temperature range were performed to obtain integrated intensity changes with temperature change. These were performed with rising temperature, and data were collected for about 10 min per point. The intensity changes indicate when motions on the time scale of the spectrometer appear.

## Results and Discussion

**Figure 1:**
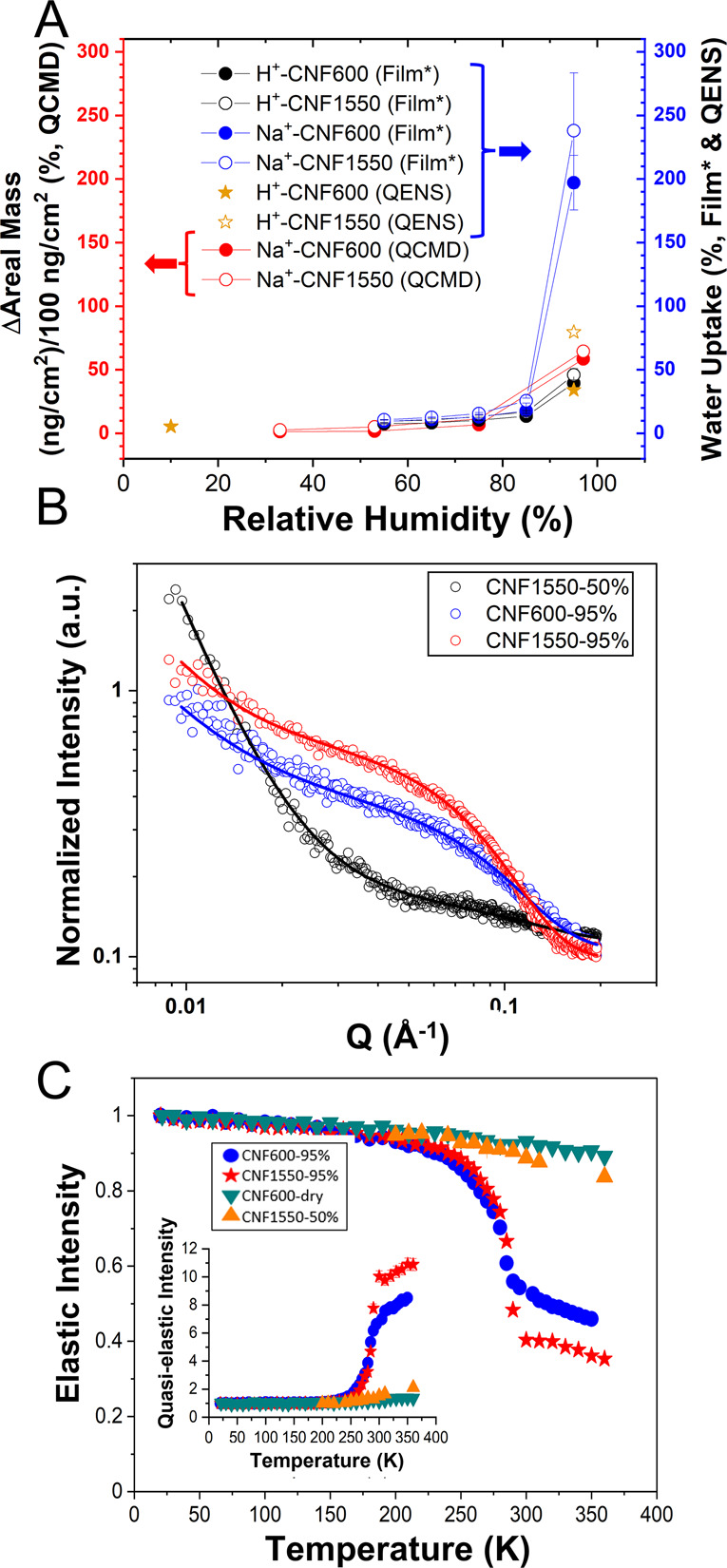
(A) Areal Mass change per 100 ng/cm^2^ (%) of the Na^+^-CNFs measured by QCM-D compared to the areal mass at RH 11%; the water uptake from the film of Na^+^-CNF and H^+^-CNF prepared via the same protocols (reproduced from Table S1 of ref (11)) and water uptake in the stacked film used for QENS; (B) morphological changes of the protonated films of CNF1550-55% (black), CNF1550-95%(red), and CNF600-95%(blue) illustrated by SAXS; (C) elastic intensity plotted against temperature for CNF600-95% (blue), CNF1550-95% (red), CNF600-Dry (dark green), and CNF1550-50% (orange). Intensities at different temperatures are integrated from 0.45 to 1.2 Å^–1^ and within the energy range of ±17.5 μeV, i.e., the so-called elastic window scan of QENS, and are normalized to the intensity at 20 K. The inset shows the intensity changes in the quasi-elastic range integrated between 0.1 and 0.3 meV.

The water uptake depends on the CNF surface charge and relative humidity (RH); higher surface charge leads to bigger water uptake with the increase of RH. In Figure 1, the results of QCM-D, SAXS, and the elastic-window scan of QENS obtained from our hydrated films are summarized to depict some general physical parameters relevant to water sorption in the films. Figure 1A collects the QCM-D areal mass change per 100 ng/cm^2^ (%) of Na^+^-CNF600 and Na^+^-CNF1550 samples due to adsorbed water with a reference areal mass measured at 11% RH (Table S2), the water uptakes of both Na^+^-CNF and H^+^-CNF from the same preparatio as performed in ref (11) (Table S1) and the water uptake of those stacked films used for QENS. For all material formats, the vapor sorption of CNF1550 is higher than that of CNF600 due to the bigger surface charge within the RH range. The difference of the areal mass change between the Na^+^-CNF1550 and Na^+^-CNF600 measured by QCM-D increases from ∼1.3 to 5.9% with the increase of RH from 33% to 97% (Table S2). For the film, Na^+^-CNF could uptake 5 times more water than H^+^-CNF at RH 95%. As mentioned before, H^+^-CNF is difficult to prepare into a well-separated single nanofibril coating on the QCM-D sensor surface without fibril aggregates. Thus, a rough estimation of the water uptake of a single H^+^-CNF nanofibril by a simple linear scaling would be ∼12.9% for CNF1550-95% and ∼11.7% for CN600-95%. The general difference between the estimated water uptake of a single H^+^-CNF nanofibril and that of measured water uptake of H^+^-CNF films is over 20%. Such estimation suggests that the film’s morphology plays a crucial role in the absorption of additional water between the fibrils. Figure 1B shows SAXS patterns of CNF1550-95%, CNF600-95%, and CNF1550-50%. With the increase in RH from 50 to 95%, the CNF films swell due to the water uptake, showing an enhanced intensity at a high *Q*, which is absent when the RH is 50%. As the intensities are all normalized to the Porod invariant as stated in the experimental part, the structure features are highlighted and are comparable. The higher scattering intensity of CNF1550-95% accounts for the higher water uptake compared to CNF600-95%, which is consistent with the moisture content. Ultimately, the CNF films equilibrated at 95% RH generate more nanostructures than those conditioned at 50% RH, which is related to the swelling of the films induced by the adsorption of water leading to the formation of channels and/or pores.^11^ The SAXS data could be reasonably fitted by a linear combination of a power law function and a generalized Guinier function (eq 4).
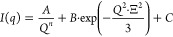
4

In this expression, *A* and *B* are scaling factors, and *C* is the background. The fitting parameters are listed in Table 1. The power law function depicts the network connection by the exponent (*n*) in the low *Q* region (<0.03 Å^–1^). At high RH, CNF1550-95% and CNF600-95% have exponent values of around 2.1 and 1.7, respectively, compared to 2.9 for the lower RH sample CNF1550-50%. This suggests that the nanofibril network turns from a massively connected network (known as mass fractal structure) into a less interconnected structure with a dominant branched fiber shape. The Guinier function reasonably reproduces the higher intensity features at high *Q* region (0.03 Å^–1^ < *Q* < 0.2 Å^–1^) at 95% RH, which are generally assigned to the average radius of gyration (Ξ) of water uptake-induced nanostructures. By comparing Ξ values of the three different samples, we found that the higher the moisture content, the larger the Ξ values. It is worth mentioning that the CNF films at RH 95% are still intact and with reasonably good mechanical properties. Despite water uptake having introduced nanostructures with radius of gyration from 1.5 to 2.1 nm, the film is still very compact. Furthermore, the radii of gyration in the three films are smaller than the nanofibril cross section. We shall interpret that these nanostructures are between nanofibrils. By assuming that these nanostructures are well-dispersed in the system, the Guinier function intensity could be approximated as .^62^ Thus, the *B* factor of eq 4 may be interpreted as a value proportional to the square of the volume of the formed nanostructures. In this case, these nanostructures in CNF1550-95% have taken up 1.38 times (i.e., ) more volume than those in CNF600-95% and 3.24 times more volume than those in CNF1550-50%. It should be noted that the hydration layer adjacent to the fibril surface may also change the thickness, which could not be easily modeled from the SAXS data. Nevertheless, the mobile water molecules should be preserved between the fibrils.

**Table 1:** SAXS Fitting Parameters in Eq 4

sample	*A*	*n*	*B*	Ξ	*C*	χ^2^
CNF600-95%	0.009	1.730	0.264	1.841	0.105	6.549
CNF1550-95%	0.005	2.112	0.506	2.102	0.098	8.823
CNF1550-50%	0.002	2.901	0.048	1.432	0.114	4.134

After characterizing the RH-dependent CNF–water interaction at the single nanofibril level by QCM-D and the water-induced nanostructure in the CNF network, we investigated the corresponding temperature dependency of water dynamics in H^+^-CNF films via QENS. The temperature range was identified by performing an elastic window in the intensity range ± 17.5 μeV by QENS over a wide temperature range from 25 to 350 K. Figure 1C shows the elastic window. Below 220 K, all the samples show similar trends where the water molecules are frozen and/or bound firmly. The observed smooth reduction in intensity due to the Debye–Waller factor, which denotes the reduction of elastic scattering intensity due to inelastic events, e.g., by thermal motion, was observed. This intensity decrease may also originate from the motions of the hydroxyls groups of the CNF. Between 220 and 270 K, the elastic intensity of the highly hydrated CNF600-95% and CNF1550-95% drops, which also translates to a set-in of quasi-elastic intensity (inset in Figure 1C). This observation suggests that water molecules gain enough thermal activation energy to become mobile. In contrast, the sample with the lower hydration CNF1550-50% shows only a small change in intensity across the temperature range comparable to the dried sample CNF600-Dry. As suggested in Figure 1A,B, the amount of water present in the CNF network at RH 50% may still be low and bound to the surface of the nanofibrils and not forming a liquid water layer within the CNF films, thus not being able to generate enough diffusion movement on the time scale of the spectrometer. This phenomenon is also reflected in the SAXS characterization with a very low intensity of the Guinier contribution (Figure 1B). Such observation is in line with our previous work,^11^ where the SAXS patterns of similar CNF films have no structural changes below 70% RH, meaning that water tends to hydrate the nanofibril’s surfaces without opening up the space between nanofibrils. After 270 K, the water molecules, which are more easily thermally activated, become completely mobile when different water dynamics take place, shown as the flatter development of the elastic and quasi-elastic intensity at higher temperature in Figure 1C. Interestingly, despite the slow increase in the quasi-elastic intensity as temperature increases in this range, the ratio between the quasi-elastic intensity of CNF1550-95% and that of CNF600-95% is rather constant around 1.4 (Figure S6) and 5.2 to CNF1550-50%. As the quasi-elastic intensity is also proportional to the amount of mobile water in the system, the value of 1.4 matches well with the Guinier intensity ratio (1.38) of those two systems. Thus, one may suggest that the Guinier-function-depicted structures be an important source of the mobile water, if not dominant. However, the value of 5.2 is larger than the Guinier ratio of 3.24 for CNF1550-95% and CNF1550-50%, indicating that mobile water may come from other sources. As Perkins and Batchelor have already pointed out, there may not be a rigid separation between mobile and immobile water as the hydration layer around the cellulose becomes thicker.^48^ It can be expected that the hydration layer is thicker at RH 95% than at 50%, so the top hydration layer may be easier to activate into a mobile state. Overall, we may interpret the temperature-dependent water motion based on the moisture content and film morphology as such: at 95% RH, the water molecules do not only hydrate the CNF surface but also open the internanofibril space. The water between the nanofibrils is easier to be mobile upon thermal activation, and the water hydrating the CNF surfaces becomes activated and mobile at higher temperature gradually from the outer hydration layer to the inner hydration layer.

**Figure 2:**
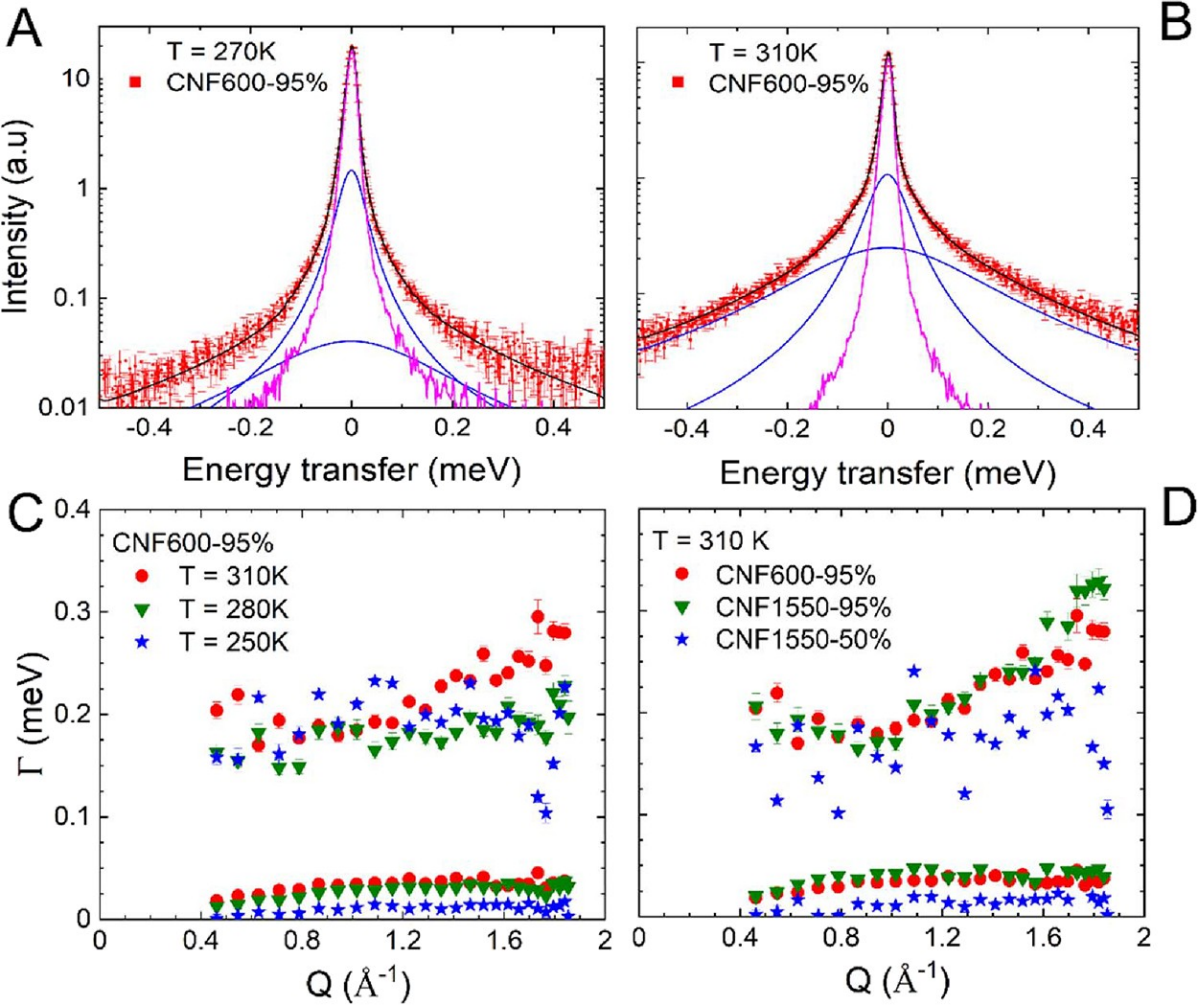
QENS spectra of CNF600-95% (*Q* vector of 1.0 Å^–1^) at 270 (A) and 310 K (B) presented using a logarithmic scale. Included are experimental data (in red) and the total fit curve (in black) via the fitting model (eq 2): the elastic contribution (in pink) and the two Lorentzian line shapes (in blue). (C) HWHM from the fits with the Lorentzian curves plotted for three different temperatures, i.e., 310, 280, and 250 K, of CNF600-95%. (D) Widths of the Lorentzian fits plotted for the three different samples, i.e., CNF600-95% (red), CNF1550-95% (green), and CNF1550-50% (blue) at the same temperature *T* = 310 K.

In the following, we will examine in detail this evolution of the quasi-elastic intensity with temperature. The quasi-elastic intensity change above 270 K is showcased by inspecting the spectra of the hydrated CNF600-95% sample at Q = 1.0 Å^–1^ (Figure S2). The QENS signal is modeled via eq 2, which consists of two Lorentzian functions, and in Figure 2A,B, we present examples of the fits. This assumption is supported by the results of the dynamic susceptibility (Figure S4), which indicate two different dynamic processes of water within the CNF films.

Figure 2A,B shows two exemplary spectra with the fitted elastic and Lorentzian contributions. This fitting perfectly describes the line shape, and there is no evidence indicating that a more sophisticated model should be considered, which is also supported via dynamic susceptibility (Figure S4). The two Lorentzians have quite different widths and describe a slower motion (the narrower width component) and a faster motion (the wider width component) of the water molecules. Such an assignment is based on the complementarity between the lifetime (τ_l_) and the spectral width. More detailed information about the nature of these two relaxation processes can be provided by analyzing the broadening of the HWHM (Γ) as a function of the wave vector. In Figure 2C, the results for three different temperatures for the HWHM of the two Lorentzians are plotted against the momentum transfer *Q*. Please note that the outliers around *Q* ≈ 1.7 Å^–1^ are related to the scattering geometry of the plate sample holder. There is a large difference in the HWHM and hence in the relaxation dynamics of the two motions. The smaller width indicates a reduction toward small *Q*, which is evidence for a translational diffusion.^51,63−66^ The broader Lorentzian however does not converge to zero width at small *Q* vectors and hence indicates a localized motion of the particles.^63,66−68^Figure 2D compares the HWHM of different films observed at *T* = 310 K as a result of water uptake. Note that the HWHM values of CNF600-95% and CNF1550-95% are very similar for both Lorentzian contributions. Apparently, the sample with a lower hydration shows a reduced mobility, which agrees with the observation in the elastic window scans (Figure 1C). The large HWHM of both highly hydrated samples indicates toward larger *Q* vectors a slight increase with *Q*, indicating a further change in mobility. Overall, the HWHM of QENS suggests that single water molecules follow similar physical diffusion mechanisms when the system is hydrated regardless of the surface charge. When the film is not sufficiently hydrated as in the case of CNF1550-50%, the motions of water molecules are rather reduced. However, it is notable that the difference in the surface charge can result in a significant difference on water uptake (Figure 1). Clearly, given the same dry weight content, CNF1550 may contain more bulk-like water. Consequently, CNF1550 could transport more protons than CNF600 per unit weight. This is also reflected in Figure 1C as the decreasing trend of elastic intensity is similar for both CNF1550 and CNF600, but CNF1550 reached a lower intensity value. The result coincides with the proton conductivity measurement of a similar kind of CNF membrane tested in a fuel cell device.^11^ Thus, for any applications of CNFs, which require water as transport media, tuning the surface charge may provide an approach to reduce the material consumption without compromising the transport properties.

As mentioned before, NMR studies suggest that water diffusion in cellulose fiber and CNFs consists of two components, which are related to mobile or less mobile water molecules. To correlate our QENS observation to the NMR characterization, we interpret that the translational motion may be correlated to the mobile water reported by NMR while the localized motion may be associated with the less mobile water.

### Translational Motion

At first, we will consider the translational water motions. Usually, a translational diffusive process indicated by the dispersive relation of HWHM as a function of *Q*^2^ can be and has been successfully described by the so-called random jump diffusion model for disordered systems.^69^ From eq 5, the diffusion coefficient *D*, and the residence time τ can be extracted. Herein, an exponential distribution of possible jump lengths is assumed to which the particle can jump after an average residence time τ. The model predicts the following form for the HWHM in dependence of the wave vector *Q*:
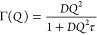
5

Toward small *Q* vectors, this model converges to the Fick diffusion model Γ = *DQ*^2^.

**Figure 3:**
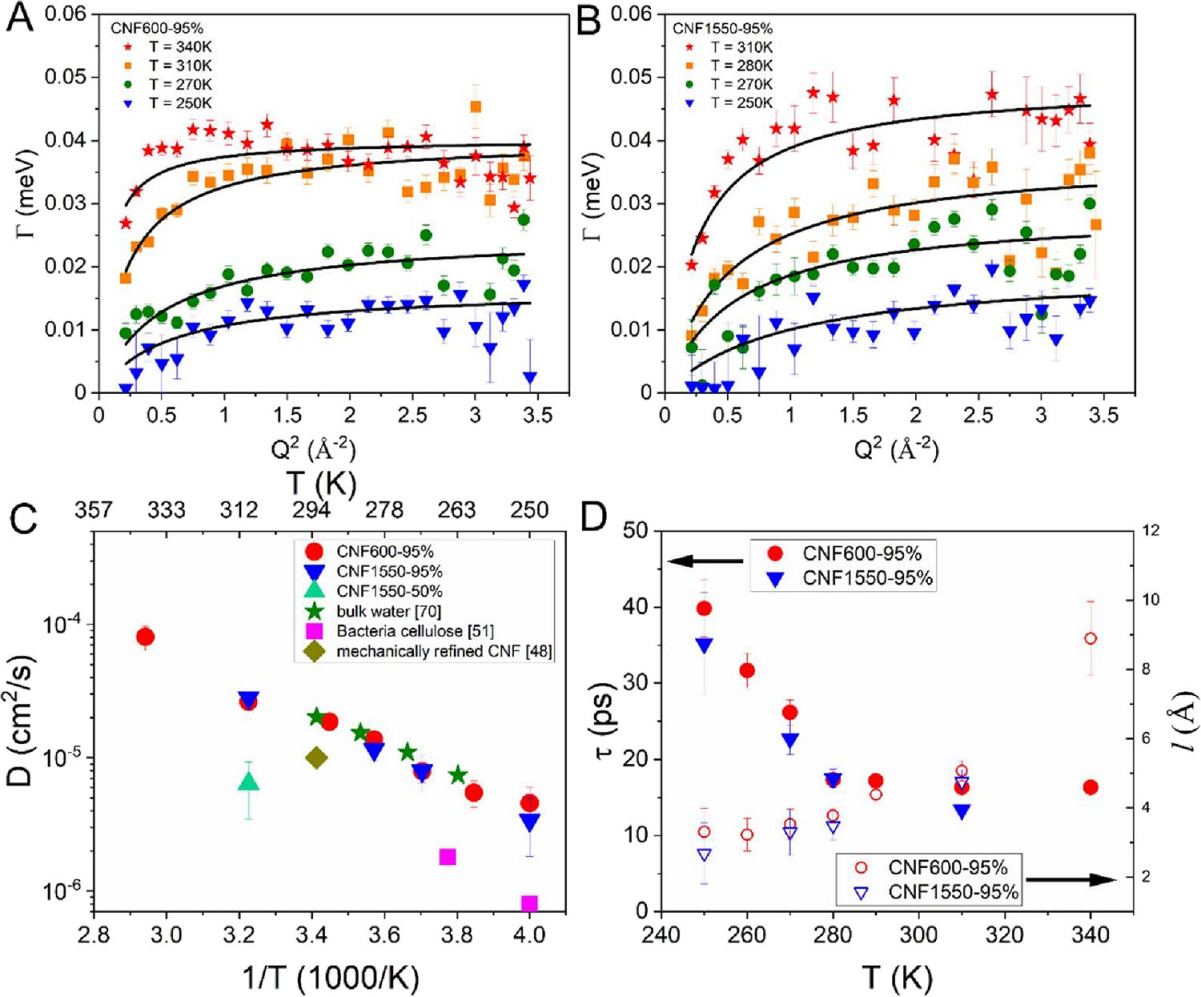
Widths of the translational motions are plotted for the CNF600-95% (A) and CNF1550-95% (B) samples for different temperatures. The lines are fits with the random jump diffusion model. (C) Resulting diffusion coefficients (*D*) are plotted in an Arrhenius-type representation and compared to values from the literature. (D) Residence times τ and jump length *l* from the random jump diffusion fits are plotted against temperature.

Figure 3A,B shows that the random jump diffusion model reasonably well reproduces the *Q*^2^-dependent HWHM for both CNF600-95% and CNF1550-95%. The resulting diffusion coefficient *D* is plotted in Figure 3C against the inverse temperature in an Arrhenius-type plot. The values of the diffusion coefficients of the samples (Figure 3C) are very similar for both surface charges and seem to align on a line in this representation, indicating an Arrhenius-type process for the translational motion. A direct comparison of the HWHM of CNF600-95% and CNF1500-95% at 250 and 310 K also shows a marginal difference and thus supports the similar diffusion coefficients (Figure S3)

Measurements with the reduced hydration CNF1550-50% delivers a diffusion coefficient *D* = 6.4 × 10^–6^ cm^2^/s at 310 K, whereas we obtain a *D* = 2.8 × 10^–5^ cm^2^/s for the CNF1550-95% sample, hence about five times smaller than the highly hydrated ones. The value for bulk water is very similar to the *D* for the hydrated CNF samples,^70^ meaning that on the nanoporous scale, the water moves like bulk water. For bulk water, *D* = 2.0 × 10^–5^ cm^2^/s at 293 K was reported,^70^ which compares quite well with *D* = 1.86 × 10^–5^ cm^2^/s for 290 K of the CNF600-95% sample. Included also are two values from a QENS study on highly crystalline bacterial cellulose, which are more similar to the reduced hydration sample.^51^ For the crystalline cellulose at 250 K, a *D* = 0.8 × 10^–6^ cm^2^/s was reported^51^ and we calculate at 250 K for the CNF600-95% sample a *D* = 4.6 × 10^–6^ cm^2^/s. By using the Arrhenius equation, the water activation energy is determined to be 20.4 kJ/mol for CNF600-95% and 21.9 kJ/mol for CNF1550-95%. These values are higher than that of water diffusion, which is about 14.6 kJ/mol, indicating that extra energy is needed to activate water diffusion in CNFs. Our derived diffusion coefficients are similar to those for bulk water, hydrated Nafion,^71^ and perfluorinated sulfonic acid (PFSA)^72^ membranes. This similarity in *D* indicates that CNFs could be applied in fuel cells, and promising tests have been performed at both a lower temperature of 30 °C^11^ and higher temperature above 100 °C.^43^ Our QENS characterization covers 30 °C and shows that the water diffusion coefficient keeps increasing up to 340 K. According to the high temperature application reported by Bayer et al.,^43^ the ion conductivity of the CNF membrane kept increasing till 100 °C before it started dropping. Thus, we may also anticipate that the water diffusion coefficient would continue to increase after 340 K. Furthermore, the *D* values at 250 and 270 K are higher than those determined in bacterial cellulose with good crystallinity,^51^ indicating that nanofibril networks may have less restriction to the water movement, which may stem from the structure between nanofibrils and was modeled via the Guinier function in SAXS. Certainly, compared to the microfibril aggregates or cellulose fibers where water molecules are trapped between the packed CNFs, water between randomly connected nanofibrils should be more mobile. Vice versa, water molecules could sufficiently lubricate the CNF motions in hydrated films, which could be one of the reasons for the known poorer wet strength of the non-cross-linked CNF film.^36^

The resulting residence times τ are shown in Figure 3D. Up to ∼280 K, τ is decreasing and hence is driving the increase in the diffusion coefficient. However, around 280 K, the dynamics seem to change, showing a constant residence time combined with an increasing jump length (). At higher temperature, the increasing diffusion constant is driven by an increasing jump length shown in Figure 3D. The jump length is around 3–4 Å below 280 K, which is slightly larger than the diameter of a water molecule ∼2.8 Å.^73^ Thus, one may interpret that the water molecules that participate in the translational diffusion first jump more frequently with a similar jump length when the temperature increases till 280 K and afterward they jump with a larger jump distance.

### Localized Motion

As shown in Figure 2C, below ∼280 K, the HWHM (Γ) of the broader Lorentzian component of the water motions in CNF-600-95% is nearly independent of the momentum *Q* transfer and indicates a purely localized movement. However, with increasing temperature, the width broadens and even increases with *Q*, which is most apparent at *T* = 340 K. Full QENS spectra at other temperatures can be found in Figure S5. A localized motion is always entangled with an elastic contribution in the spectra.^69^ This elastic contribution to the total spectrum is called the elastic incoherent structure factor (EISF), which is obtained experimentally according to eq 6. It is the long-time limit of the intermediate scattering function, which is directly related to the region of space accessible to a scatterer and describes the geometry of the localized motion for a single incoherent scattering particle.^69^ The analysis of the EISF will therefore provide insight into the localized motion of the hydrogens. In order to obtain the pure motion of the moving water molecules, the elastic incoherent scattering from the cellulose needs to be separated. This was achieved by subtracting the elastic intensity of the dry sample taken at *T* = 250 K from the fitted elastic intensity of the hydrated samples:
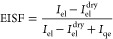
6

**Figure 4:**
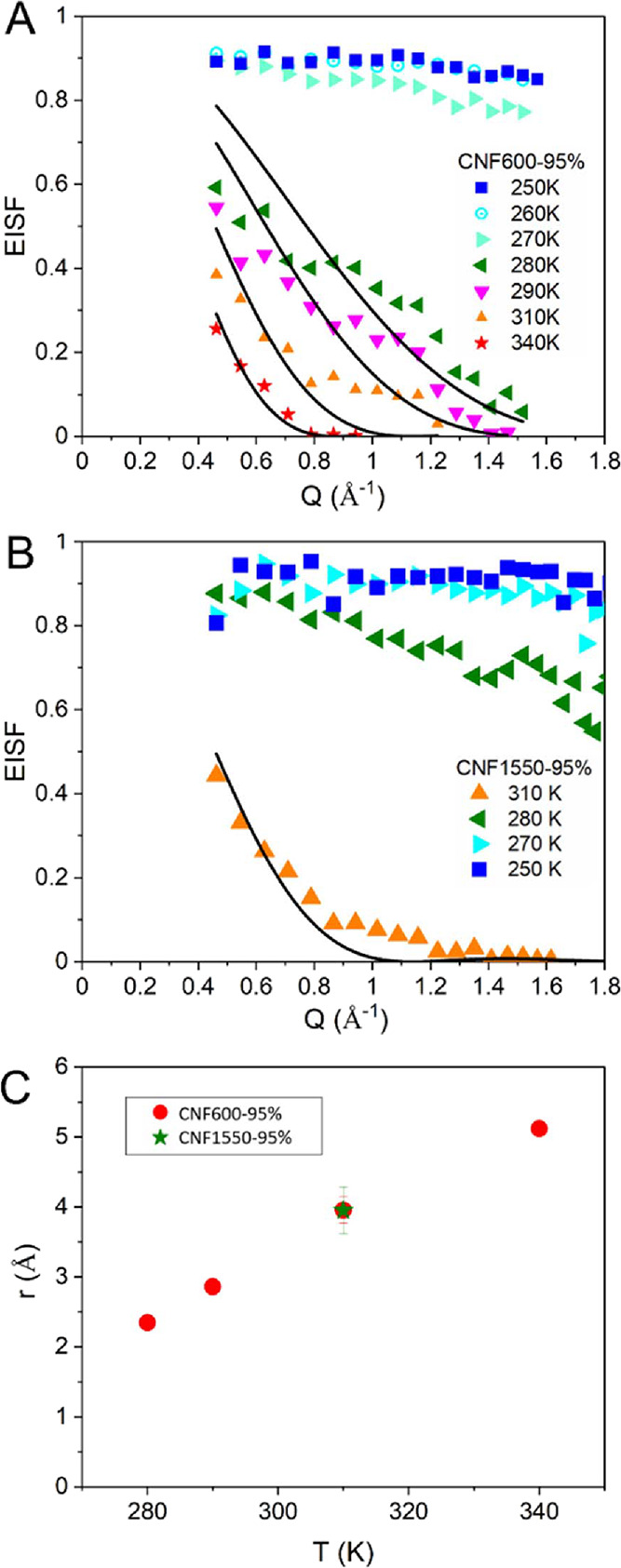
(A) EISFs for CNF600-95% and (B) for the CNF1550-95% sample plotted for all measured temperatures. Included as lines are fits with a model comprising the diffusion inside a sphere. (C) Plot of the derived radius of the confinement sphere from the fit to the EISF model.

Figure 4A,B plots the resulting EISF of the CNF600-95% and CNF1550-95% samples. There is a distinct change in dynamics around 270 to 280 K. Below this temperature range, the EISF does not decrease very much with increasing *Q*. This *Q* dependence of the EISF could be described by a localized jump of frozen water molecules over a short distance. However, a dramatic change in dynamics occurs with rising temperature in the same temperature range where we observe a change in the temperature behavior of the residence time τ of the translational diffusion. In the CNF with the larger surface charge, the motion changes at slightly higher temperatures and it might be because the surface charge influences the mobility of the water molecules. To learn more about the geometry of the localized movement at higher temperature, modeling of the EISF is in order. It is notable that the derived EISFs at high temperature approach zero within the observed wave vector range. Such behavior agrees with the predictions from the scattering law for diffusion in a spherical potential within a sphere of a specific radius *r*. The expected EISF for such a motion is given by Volino et al.:^74^
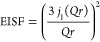
7with *j*_1_(*Qr*) is the spherical Bessel function of the first order, which is  .

Equation 7 was fitted to the experimental EISF data above 280 K. Note that the EISF is always converging to 1 toward *Q* = 0 and deviations might occur due to multiple scattering not being corrected. The fit qualitatively reproduces the development trend. From the fit to the EISF, a radius of the confinement sphere can be extracted. Figure 4C plots the resulting radius values. From *T* ≈ 280 K onward, the radius increases dramatically and indicates a freer movement of the water molecules within a sphere of increasing dimensions. The change in the geometry is also reflected in a change in the *Q* dependence of the width (Figure 2C,D) at elevated temperature, i.e., from a constant width up to about *Q* ≈ 1 Å^–1^, the HWHM increases towards larger Q. A diffusion-like movement of the particles is expected within the confinement sphere.^74^ The model predicts not only a certain *Q* dependence for the EISF, which we have used to fit our data, but also toward larger *Q* an increase of the width, reflecting the diffusive type of motion on short length scales. The crossover between localized and diffusive motion is predicted to be around (*Qr*)^2^ ≈ 10.^74^ With a radius of around *r* ≈ 3 Å and *Q* ≈ 1 Å^–1^, where the width sets in to increase, that prediction might quite be well-fulfilled in our case. Note that the water diameter is about 3 Å and hence the confinement space is at least twice the size of a water molecule.

Finally, we would like to connect the localized motion and the translational motion to the whole picture of the water dynamics captured by QENS: The elastic intensity drops quickly from 250 to 280 K, where we observe a residence time change for water molecules participating in the translational motion. The ratio between the elastic intensity decreases in CNF600-95% and that in CNF1550-95% matches well with the ratio between the amount of interfibril structure in CNF600-95% and that in CNF1550-95% upon water uptake as shown by SAXS. It may suggest that such internanofibril water molecules contribute to translational diffusion, which had already been proposed by NMR studies.^48^ Meanwhile, the localized motion indicates a dramatic change around 280 K from a localized jump to a confined motion, which can be described by a diffusion inside a sphere. A picture emerges that a fraction of all water molecules participates in translational diffusion down to the lowest resolvable temperatures. This water is still mobile below 270 K. Second, there exists a fraction of water molecules, which is less mobile on the CNF at temperatures below 280 K and which performs diffusive motion within a confined space above this temperature. At this moment, it is still difficult to assign where the localized motion exactly occurs because of the controversial discussion of the crystalline and non-crystalline distribution along the CNF. Nevertheless, we suggest that water molecules closer to the CNF surface may be more constrained because of the enthalpic dominant adsorption mechanism. As previously pointed out by Topgaard and Söderman, at the surface, the cellulose–water interaction may be influenced by different interactions at the atomic scale. The fit could certainly be improved by introducing more sophisticated models, which require a dedicated study in the future.

## Conclusions

We combined QCM-D, SAXS, and QENS to investigate the water dynamics in hydrated CNF films with carboxylated surface ligands. Such water dynamics are closely related to the proton conductivity, which is important for fuel cell applications. QCM-D and SAXS suggest that water uptake should not only hydrate the CNF surface but also open the internanofibril distance by forming nanostructures. The higher the surface charge of the CNF has, the more nanostructures are formed. The QENS spectra indicate two types of motions for the water molecules: a localized motion and a translational diffusion. The fraction of water molecules participating in translational diffusion may be located between CNFs and first get activated upon increasing the temperature from 250 K on the time scale of the spectrometer. The determined diffusion coefficient is very close to bulk water and that of the Nafion membrane above room temperature. The less hydrated sample shows a factor of five reduced diffusion coefficient. The amount of charge on the surface does not influence the translational mobility. Furthermore, we observe that a fraction of water molecules evidences a localized motion. The localized motion shows a strong change above 280 K, and the analysis of the EISF provides a qualitative picture that the motion can be described as a diffusion in a sphere with an increasing radius with increasing temperature. By focusing on the translational diffusion, the higher surface charge of CNF1550 at 95% RH allows it to carry twice as much water molecules as CNF600, but at the same time, the diffusion coefficients are similar from an experimental perspective in both systems. The fact that the amount of water molecules able to carry the proton in the CNF1550 samples is twice that in CNF600 accounts for examples for the difference in proton conductivity or ionic transport in actual applications.

## Supporting Information Available

QCMD characterization; water uptake of H^+^-CNF and Na^+^-CNF reproduced from ref (11); areal mass and water uptake of QCM-D; QENS resolution and quasi-elastic intensity change; CNF600-95% QENS profile at *Q* = 1.0 Å^–1^; comparison of HWHM of the slow translational diffusion of CNF600-95% and CNF1550-95% at 250 and 310 K; full set of dynamic susceptibilities of CNF600 and CNF1550; HWHM of the fast local movement of CNF600-95% and CNF1550-95% at different temperatures; quasi-elastic intensity ratio between CNf 1550 and CNF600 at RH 95%(PDF)
