# Supplementary material for: The Impact of Surface Charges of Carboxylated Cellulose Nanofibrils on the Water Motions in Hydrated Films
Source: Biomacromolecules. 2022 Jul 5;23(8):3104–15. doi: 10.1021/acs.biomac.1c01517 (PMC9364319; doi:10.1021/acs.biomac.1c01517)
Supplement: Supplementary file 1 — bm1c01517_si_001.pdf [file bm1c01517_si_001.pdf]

Supporting Information

The Impact of Surface Charges of Carboxylated Cellulose Nanofibrils on The Water Motions in The Hydrated Films

Valentina Guccini[1,2], Shun Yu[1,3,], Zhoujun Meng[2], Eero Kontturi[2], Franz Demmel[4], Germán Salazar-Alvarez[1,5,6*]*

1. Department of Materials and Environmental Chemistry (MMK), Stockholm University, SE-10691, Stockholm, Sweden
2. Department of Bioproducts and Biosystems, School of Chemical Engineering, Aalto University, P.O. Box 16300, 00076 Aalto, Finland
3. Smart Materials, Division of Bioeconomy and Health, RISE Research Institute of Sweden, Drottning Kristinas väg 61, 114 86 Stockholm, Sweden

[*] Corresponding authors: Shun Yu shun.yu@ri.se and Germán Salazar-Alvarez german.salazar.alvarez@angstrom.uu.se

4. ISIS Facility, Rutherford Appleton Laboratory, Didcot OX11 0QZ, UK

5. Department of Materials Science and Engineering, Ångström Laboratory, Uppsala University, Box 35, SE-751 03, Uppsala, Sweden

6. Center for Neutron Scattering, Uppsala University, Box 35, SE-751 03, Uppsala, Sweden

QCMD Characterization. After the coating, the surface of CNFs coated QCM-D sensor was imaged by atomic force microscopy (AFM) to confirm the individualized CNFs coated on the surface. Figure S1 presents two CNFs on the sensor surface indicated by the arrows. The table shows the saturated salts solutions and MilliQ which are used to regulate the relative humidity inside the QCM-D modulus during the water vapor absorption measurements.

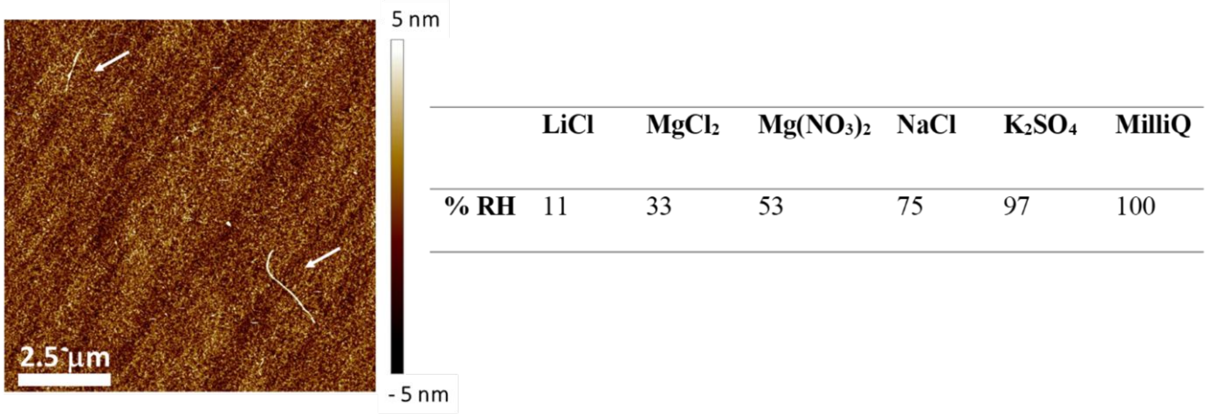

	LiCl	$MgCl_2$	$Mg(NO_3)_2$	NaCl	K_2SO_4	MilliQ
**% RH**	11	33	53	75	97	100

Figure S 1 (A) AFM image of CNFs coated QCM-D sensor. The CNFs are indicated by the two arrows. The table shows the Relative Humidity of the saturated salt solutions pumped through the QCM-D humidity modulus.

Table S1 water uptake of the CNF membrane. Reproduction from *V. Guccini, A. Carlson, S. Yu, G. Lindbergh, R. W. Lindström and G. Salazar-Alvarez, J. Mater. Chem. A, 2019, 7, 25032* ***DOI:*** *10.1039/C9TA04898G*

Relative Humidity (%)	H-CNF-600	H-CNF-1550	Na-CNF-600	Na-CNF-1550
55	7.5 ± 0.4	9.5 ± 1.1	9.4 ± 1.4	10.6 ± 0.3
65	8.3 ± 0.4	10.4 ± 1.0	10.9 ± 1.5	12.5 ± 0.2
75	10.4 ± 0.2	13.4 ± 0.8	13.0 ± 1.4	15.6 ± 0.6
85	13.5 ± 0.4	16.7 ± 1.0	17.7 ± 2.9	25.7 ± 1.9
95	39.5 ± 2.6	45.9 ± 4.9	197.0 ± 21.5	237.9 ± 45.4

Table S1 shows the water uptake of the CNF membranes at different relative humidities reproduced from the Table S1 of Guccini et al. *J. Mater. Chem. A, 2019, 7, 25032* ***DOI:*** *10.1039/C9TA04898G.*

Having sodium as counter ion or being a protonated carboxylate group, affects the absolute value of water uptake, but not the general behaviour of the films. below 80 % RH the vapor uptake of the film CNF1550 with sodium and hydrogen counterions is not significantly different, at 85 % RH still extremely similar and at 95 % RH the difference is of 1 order of magnitude. This means that despite the vastly different in pH outside and inside the films of the protonated ones, the vapor uptake changes dramatically compare to the films with sodium counterions only above 90% RH. The same trend can be seen at the nanofibril level.

Table S 2 Areal Mass and water uptake of Na^{+}-CNF1550 and Na^{+}-CNF600 determined by QCM-D

	Areal Mass (ng cm^{-2})				
	$LiCl$ (11% RH)	$MgCl_2$ (33% RH)	$Mg(NO_3)_2$ (53% RH)	$NaCl$ (75% RH)	K_2SO_4 (97% RH)
**1550-CNF**	508	521,7	534	566,4	835,3
**ΔAM**	-	13,7	26	58,4	327,3
**AM_{water}/100ng**	-	2,7	5,1	11,5	64,4
**600-CNF**	489	495,9	497,4	531,4	775,2
**ΔAM**	-	6,9	8,4	33,4	286,2
**AM_{water}/100ng**	-	1,4	1,7	6,8	58,5

QENS Characterization.

Resolution and Quasi-elastic intensity change. For the used configuration (PG002) of the IRIS spectrometer the full width at half maximum (FWHM) resolution is 17.5 μeV. Figure S2(A) shows a spectrum at Q = 1.0 Å^{-1} of CNF600-95% at 290 K on a logarithmic scale. Included is the 20K spectrum, which demonstrates the quasi-elastic intensity at higher temperatures. The empty can spectrum is so small that it was neglected in the data reduction process. The flat sample can was installed in a 45 degree transmission geometry in a cryofurnace, which controlled the temperature ± 1K. The sample geometry with respect to the beam deteriorates the data quality in a wave vector range around Q ≅ 1.7 Å^{-1}. Figure S2(B) shows spectra of the hydrated CNF600-95% sample at Q=1.0 Å^{-1}. It demonstrates that the

quasielastic intensity changes distinctly above 270 K in agreement with the elastic intensity plot in Figure 2 in the main text.

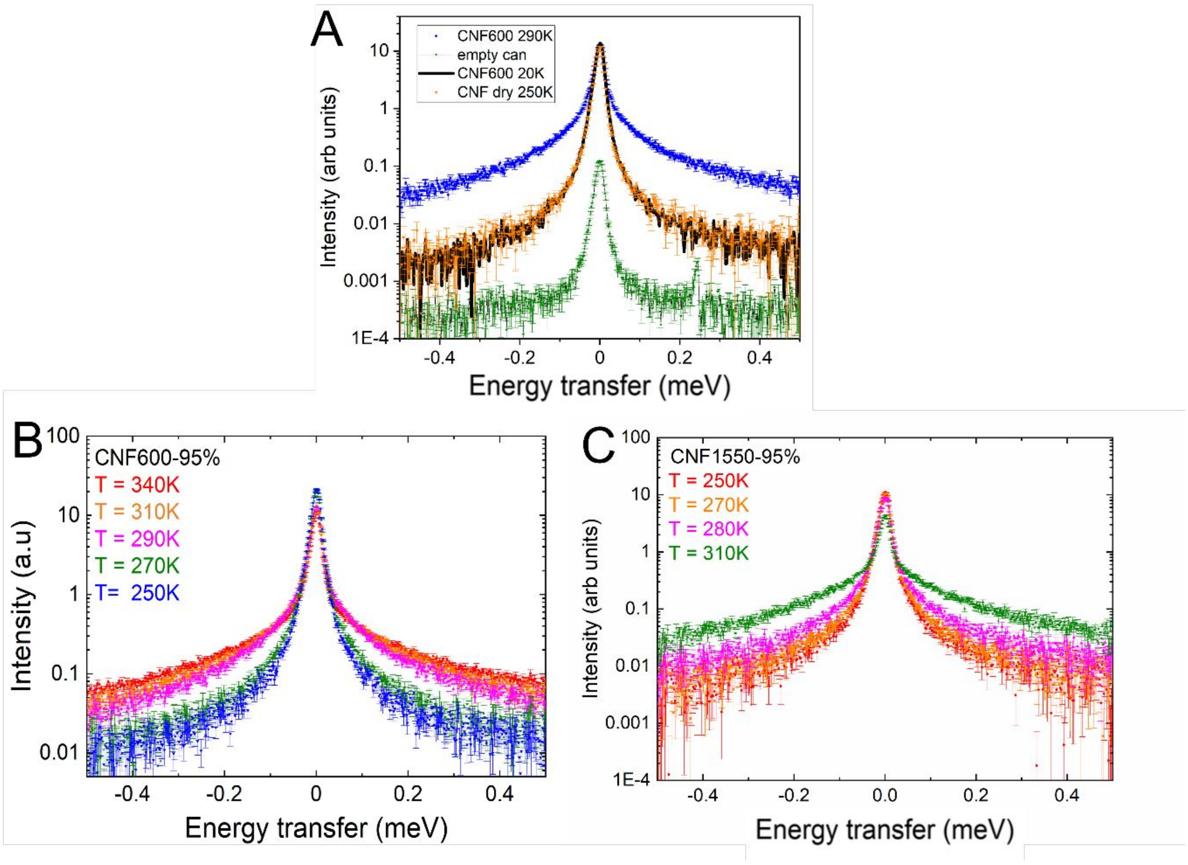

Figure S 2 (A) Spectrum of the CNF600-95% sample (blue) is plotted on a logarithmic scale at 290K in comparison to the spectrum at 20K, which represents the resolution function of the spectrometer. The 250K spectrum (yellow) shows that a part of the CNF vibrational density of States is already included in the resolution function=20K. Included is also a spectrum from the empty can, demonstrating the negligible amount of background in these measurements. **(B)** Spectra of the hydrated CNF600-95% at Q=1.0 Å^{-1}; **(C)** Spectra of the hydrated CNF1550-95% at Q=1.0 Å^{-1}.

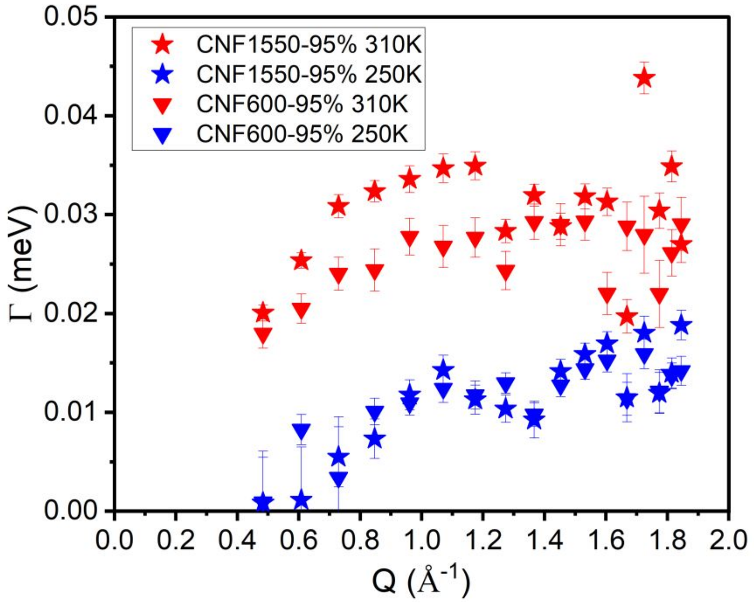

Figure S 3 shows a comparison of the slow diffusive motion from the two samples. The widths are very similar from both samples and the different surface charge has only a small influence on the dynamics of water.

Dynamic susceptibility. The dynamic susceptibility *χ´´(Q, E)* suppresses the contribution of the elastic line and enhances the inelastic features[s1–3]. It can be calculated by normalizing the QENS spectrum to the Bose population factor n_B (Eq. 5).

$$\chi''(Q,E) = \frac{I(Q,E)}{n_B(T,E)} \qquad (Eq.1)$$

Where, $n_B(T,E) = \left(1 - exp\left(-{}^{E}/_{k_BT}\right)\right)^{-1}$, E is the transferred energy, T is the temperature and k_B is the Boltzmann constant[4]. **Figure S4** shows the dynamic susceptibility of CNF600-95% and CNF1550-95% at selected temperature and water content. For both CNF600-Dry and CNF600-95% at 20 K, the water molecules are either too few or too immobile. The peak around 0.01 meV is given by the

resolution, which is not deconvoluted in the susceptibility. At higher energy transfer values (ω) two contributions become obvious for hydrated sample at a higher temperature.

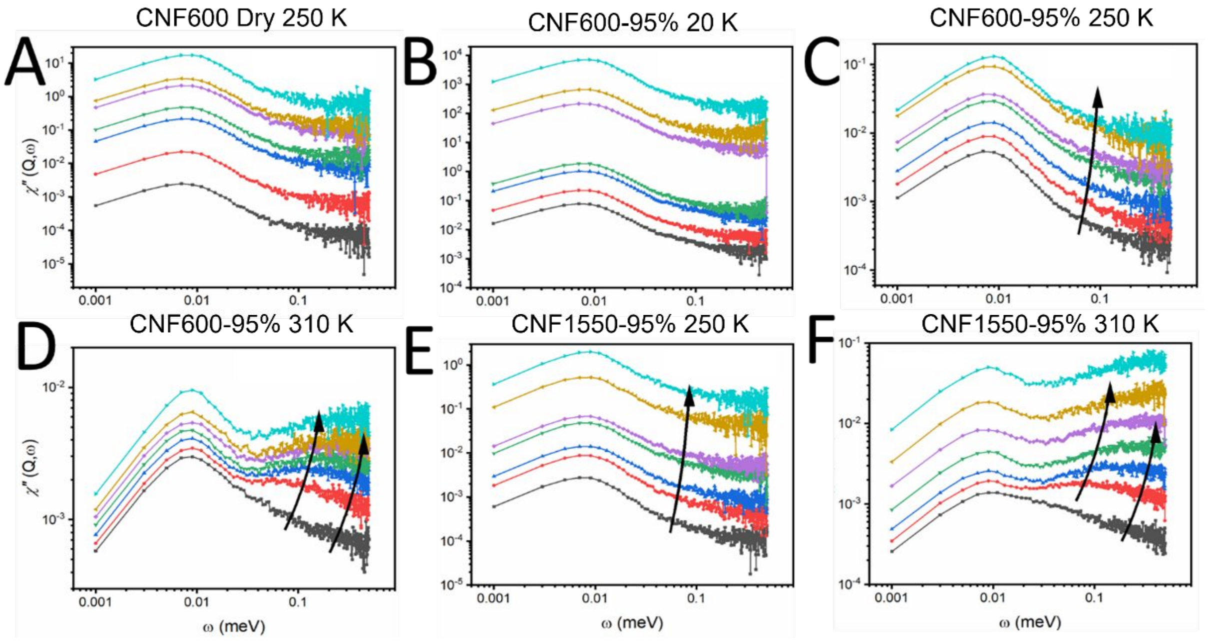

Figure S 4 Dynamic susceptibility of CNF600 Dry, CNF600-95% and CNF1550-95% at different temperatures for 7 selected transferred wavevector Q at 0.48 $Å^{-1}$ (grey), 0.84 $Å^{-1}$ (red), 1.17 $Å^{-1}$ (blue), 1.54 $Å^{-1}$ (green), 1.60 $Å^{-1}$ (purple), 1.72 $Å^{-1}$ (yellow), and 1.84 $Å^{-1}$ (cyan). The spectra were shifted vertical to highlight the dispersity. Solid Arrow guide eyes to show the dispersity.

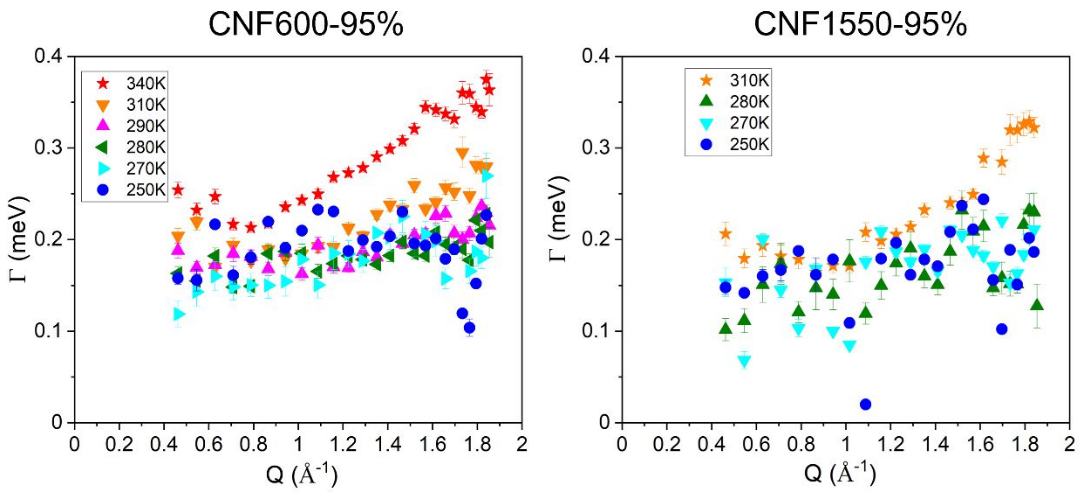

Figure S 5 The Q dependent HWHM from the CNF600-95% (left) and CNF1550-95% (right) of the broader, faster Lorentzian fit function are plotted for several temperatures.

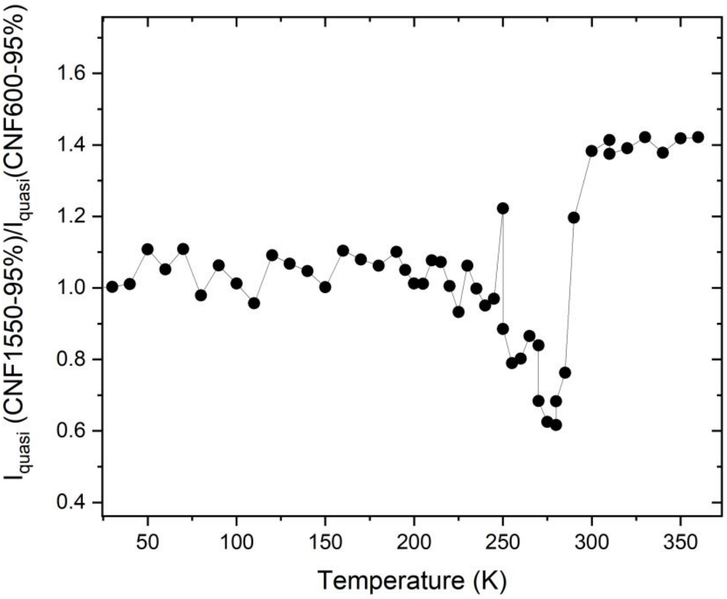

Figure S 6 quasi-elastic intensity ratio between CNF1550-95% and CNF600-95%, which is calculated by dividing their intensities of the elastic window scans.

References:

S(1) Nickels, J. D.; O'Neill, H.; Hong, L.; Tyagi, M.; Ehlers, G.; Weiss, K. L.; Zhang, Q.; Yi, Z.; Mamontov, E.; Smith, J. C.; Sokolov, A. P. Dynamics of Protein and Its Hydration Water: Neutron Scattering Studies on Fully Deuterated GFP. *Biophys. J.* **2012**, *103* (7), 1566–1575. https://doi.org/10.1016/j.bpj.2012.08.046.

S(2) Berrod, Q.; Lyonnard, S.; Guillermo, A.; Ollivier, J.; Frick, B.; Gébel, G. QENS Investigation of Proton Confined Motions in Hydrated Perfluorinated Sulfonic Membranes and Self-

Assembled Surfactants. *EPJ Web Conf.* **2015**, *83*, 02002. https://doi.org/10.1051/epjconf/20158302002.

S(3) Neill, H. O.; Pingali, S. V.; Petridis, L.; He, J.; Hong, L.; Urban, V.; Evans, B.; Langan, P.; Smith, J. C.; Davison, B. H. Dynamics of Water Bound to Crystalline Cellulose. **2017**, No. May, 1–13. https://doi.org/10.1038/s41598-017-12035-w.
